# Increased proteinase 3 and neutrophil elastase plasma concentrations are associated with non-alcoholic fatty liver disease (NAFLD) and type 2 diabetes

**DOI:** 10.1186/s10020-019-0084-3

**Published:** 2019-05-02

**Authors:** Andreea-Manuela Mirea, Erik J. M. Toonen, Inge van den Munckhof, Isabelle D. Munsterman, Eric T. T. L. Tjwa, Martin Jaeger, Marije Oosting, Kiki Schraa, Joost H. W. Rutten, Marinette van der Graaf, Niels P. Riksen, Jacqueline de Graaf, Mihai G. Netea, Cees J. Tack, Triantafyllos Chavakis, Leo A. B. Joosten

**Affiliations:** 1 Department of Internal Medicine, Radboud Institute for Molecular Life Sciences (RIMLS), Radboud University Medical Centre, Nijmegen, The Netherlands; 2 Department of Medical Genetics, Iuliu Hatieganu University of Medicine and Pharmacy, 400349 Cluj-Napoca, Romania; 3 R&D Department, Hycult Biotechnology, Uden, The Netherlands; 4 Department of Gastroenterology and Hepatology, Radboud University Medical Center, Nijmegen, The Netherlands; 5 Department of Radiology and Nuclear Medicine, Radboud University Medical Center, Nijmegen, the Netherlands; 6 Department for Genomics & Immunoregulation, Life and Medical Sciences Institute (LIMES), University of Bonn, 53115 Bonn, Germany; 7 Institute for Clinical Chemistry and Laboratory Medicine, University Hospital Carl-Gustav-Carus, TU Dresden, Dresden, Germany; 8 Paul Langerhans Institute Dresden of the Helmholtz Zentrum München at the University Hospital and Faculty of Medicine Carl Gustav Carus of TU Dresden, Dresden, Germany; and German Center for Diabetes Research (DZD e.V.), Neuherberg, Germany, Dresden, Germany; 9 German Center for Diabetes Research (DZD e.V.), Neuherberg, Germany

**Keywords:** Obesity, Inflammation, NAFLD, Type 2 diabetes, Neutrophil serine proteases, Alpha-1 antitrypsin

## Abstract

**Introduction:**

Non-alcoholic fatty liver disease (NAFLD) is becoming a major health problem worldwide. Inflammation plays an important role in disease pathogenesis and recent studies have shown a potential role for the neutrophil serine proteases (NSPs) proteinase-3 (PR3) and neutrophil elastase (NE) in NAFLD as well as an imbalance between NSPs and their natural inhibitor alpha-1 antitrypsin (AAT). The aim of this study was to investigate whether PR3 and NE plasma concentrations are associated with NAFLD and/or type 2 diabetes.

**Methods:**

To explore this hypothesis we used several cohorts: a cohort of 271 obese individuals with liver steatosis, a cohort of 41 patients with biopsy-proven NAFLD, a cohort of 401 obese type 2 diabetes patients and a cohort of 205 lean healthy controls; and measured PR3 and NE plasma concentrations. In addition, we measured AAT plasma concentrations in order to investigate if the ratios between NSPs and their natural inhibitor were altered in NAFLD and type 2 diabetes when compared to healthy controls.

**Results:**

Our data shows an increase in PR3 and NE concentrations and a decrease in AAT concentrations in obese patients when compared to controls. Moreover, PR3 plasma concentrations are increased in patients with liver steatosis. Furthermore, PR3 and NE concentrations in the liver are associated with the advanced stages of NAFLD characterized by NASH and/ or liver fibrosis. Additionally, PR3 and NE concentrations were up-regulated in patients with type 2 diabetes when compared to lean and obese controls.

**Conclusion:**

We conclude that circulating levels of NSPs associate with obesity-related metabolic disorders. Further research is needed to clearly establish the role of these proteases and investigate whether they could be used as non-invasive markers for NAFLD and/or type 2 diabetes.

**Electronic supplementary material:**

The online version of this article (10.1186/s10020-019-0084-3) contains supplementary material, which is available to authorized users.

## Background

Non-alcoholic fatty liver disease (NAFLD) has a prevalence of approximately 25% among the global population (Younossi et al. 2016), and is increasing rapidly, in parallel with the increasing prevalence of obesity. NAFLD is highly associated with the metabolic syndrome, a medical condition characterized by the combination of abdominal obesity, high blood pressure, insulin resistance and dyslipidemia (Dietrich and Hellerbrand 2014; Asrih and Jornayvaz 2015).

The disease can range from plain liver steatosis to a more severe form called non-alcoholic steatohepatitis (NASH) characterized by liver inflammation and hepatocyte ballooning, which can further progress into liver fibrosis, cirrhosis and even hepatocellular carcinoma (Machado and Cortez-Pinto 2014). Although the pathogenesis of NAFLD is not completely understood, it is known that inflammation plays an important role in disease development and progression. The pro-inflammatory cytokine IL-1β (Interleukin-1β) is one of the key cytokines responsible for induction and perpetuation of inflammation in the liver, thereby contributing to disease severity (Mirea et al. 2018). IL-1β is secreted as an inactive precursor and needs enzymatic processing in order to become bioactive. The well-known enzyme available for processing and activation of IL-1β is caspase-1, a cysteine protease activated by the NLRP3 inflammasome protein complex. In recent years, several studies have shown that caspase-1 and/or components of the inflammasome are involved in NAFLD and other metabolic disorders (Dixon et al. 2012, 2013; Wan et al. 2015; Wree et al. 2014). However, it has been reported that several other proteases are also able to process pro-IL1β. The neutrophil serine proteases (NSPs) proteinase-3 (PR3) and neutrophil elastase (NE) are able to process IL-1 β to its bioactive form independently of caspase-1-NLRP3 inflammasome complex (Mirea et al. 2018).

NSPs are anti-microbial peptides that are stored in the azurophilic granules of neutrophils. Upon neutrophil activation, they are released from the granules and can activate cytokines in the neutrophil cytosol or in the extracellular space. Outside the cell, NSPs are inhibited by alpha-1 antitrypsin (AAT), a serine protease inhibitor mainly produced by the liver (Korkmaz et al. 2010). Although it is well-known that these NSPs are involved in several inflammatory diseases, their role in metabolic diseases is less investigated. However, in recent years, several studies have shown that NE and PR3 are important drivers of chronic inflammation leading to metabolic disturbances in mouse models for obesity-induced insulin resistance, type 2 diabetes and NAFLD (Mansuy-Aubert et al. 2013; Toonen et al. 2016; Talukdar et al. 2012). Overall, these studies suggest that, an imbalance between the concentrations of NE, PR3 and their inhibitor AAT may contribute to these metabolic disturbances.

While these findings have been mainly reported in murine experimental models of the disease, little is known whether NSPs are also involved in NAFLD and related metabolic conditions in humans. The aim of this study was to investigate whether PR3 and NE plasma concentrations are associated with liver fat content and the development of fatty liver disease in a cohort of 271 obese individuals with liver steatosis diagnosed by ^1^H-MRS (proton magnetic resonance spectroscopy). Because some of these individuals had high alcohol consumption and were susceptible to develop alcoholic liver disease (ALD), another cause of fatty liver disease; we first analyzed the relation between NSPs and liver fat content in the whole cohort and further on we analyzed NSPs plasma concentrations in individuals at risk to develop NAFLD and individuals at risk to develop ALD. Moreover, to better understand the role of NSPs in NAFLD, we investigated a cohort of 41 individuals with biopsy-diagnosed NAFLD. In addition, we assessed whether circulating concentrations of neutrophil serine proteases are associated with the development of insulin resistance and type 2 diabetes in a well-characterized cohort of 401 individuals with type 2 diabetes.

## Material and methods

### Patient cohorts

Three hundred two individuals, aged 55–80, with a BMI (body mass index) above 27 kg/m^2^ were enrolled in the 300-OB study (IN-CONTROL: study of the Cardiovascular research Netherlands Project). To investigate the relation between PR3, NE and AAT plasma concentrations and the development of liver steatosis we only selected the individuals with the hepatic fat content assessed by ^1^H-MRS (*n* = 271). Because some of the included individuals had a high alcohol intake, we also divided this cohort based on the alcohol intake (European Association for the Study of the L, European Association for the Study of D, European Association for the Study of O 2016) in individuals at risk to develop alcoholic liver disease and individuals at risk to develop non-alcoholic liver disease. Furthermore, to explore the relation between PR3, NE and AAT and the advanced stages of NAFLD we selected a cohort of 41 patients with biopsy-diagnosed NAFLD. Patients included in this cohort were recruited in the context of another clinical study regarding development of fibrosis in NAFLD and patients’ characteristics were previously described (Munsterman et al. 2018).

To explore the relation between PR3, NE and AAT concentrations and the development of type 2 diabetes, 401 type 2 diabetes patients, which were part of the local Parelsnoer cohort type 2 diabetes (Navis et al. 2014) (https://parelsnoer.org), were included.

In addition, 205 lean healthy individuals were included as a control cohort for our analysis. These healthy individuals were part of the 500 Functional Genomic Project.

All projects were approved by the Ethical Committee of Radboud University Medical Center, Nijmegen. Experiments were conducted according to the principles expressed in the Declaration of Helsinki. Samples of venous blood were drawn after informed consent was obtained.

### Assessment of liver fat content

The hepatic fat content was quantified using localized proton magnetic resonance spectroscopy (^1^H-MRS) (Navis et al. 2014). A single voxel was positioned in the right lobe of the liver. The voxel was placed outside the biliary tree and blood vessels to avoid confounding of the region of interest. All images from MR spectroscopy were post-processed using the jMRUI software v3.0 package and the AMARES algorithm to determine water (4.7 ppm) and methylene (1.3 ppm) resonance areas. The frequency of the second peak was shifted between 10 to16 ppm and a phase correction between − 0.2 to 0.2 was performed. Intrahepatic triglyceride content was expressed as the area of the lipid peak by the sum of the areas of the methylene lipid peak and the water peak. The areas were not corrected for the differences in relaxation time between the water and lipid signals, as we used a short echo time (20 msec). The cut-off concentration for diagnosing liver steatosis was a total liver fat content > 5.6% (Kroese et al. 2009). Individuals with normal liver fat content (≤5.6%) were included as obese controls without liver steatosis (*n* = 105). The remaining 166 individuals had a liver fat content > 5.6% and formed the liver steatosis group.

### PR3, NE, AAT and hsCRP measurements

PR3, NE and AAT plasma concentrations were quantified using sandwich ELISAs (enzyme-linked immunosorbent assay; cat# HK384, HK319 and HK387 respectively, Hycult Biotech, Uden, The Netherlands) according to manufacturer’s instructions. PR3 concentrations were measured in plasma samples from all 271 obese individuals and 205 healthy controls. For the 401 type 2 diabetes individuals, PR3 concentrations were measured in serum samples and values were adjusted in order to be able to compare to concentrations measured in plasma samples. The adjustment was realized as follows: we measured PR3 concentrations in a subset of 12 individuals (6 from the type 2 diabetes cohort and 6 from the 271 obese cohort) for which both serum and plasma samples were available. Results showed that serum concentrations were 3 times higher when compared to plasma concentrations (Additional file 1: Figure S1). Subsequently, serum concentrations for all type 2 diabetes individuals were corrected by a factor 3 and these values were considered equivalent to plasma concentrations. NE and AAT concentrations were measured in plasma samples for all cohorts.

In order to investigate the balance between NSPs and their natural inhibitor AAT, PR3 to AAT ratio and NE to AAT ratio were calculated by dividing PR3, respectively NE plasma concentrations to AAT plasma concentrations.

Because some values for PR3, NE or AAT were outside the assay ranges, the concentrations of these proteins could not be determined in some individuals and final numbers of patients and controls included in the analysis are slightly different from the numbers initially included in the study.

High sensitive C-Reactive Protein (hsCRP) concentrations were assessed by ELISA following manufacturer’s instructions (R&D Systems, BioTechne, Minneapolis, MN, USA).

### Liver lysates measurements

Liver lysates were prepared as previously described (Munsterman et al. 2018). PR3 and NE concentrations were measured in liver lysates according to manufacter’s protocol (cat# HK384, HK319 respectively, Hycult Biotech, Uden, The Netherlands). Total protein content was measured using BCA (bicinchoninic acid protein assay) following manufacturer’s instructions (Compat-Able BCA Protein Assay Kit, ThermoFisher Scientific). Final PR3 and NE concentrations in the liver were calculated as ng PR3/mg protein respectively ng NE/mg protein.

### Statistical analysis

Statistical analysis was performed using IBM SPSS Statistics 22 (IBM, Armonk, NY, USA). The data was not normally distributed and it was natural logarithm transformed. Given that age and BMI are confounding factors for the development of NAFLD and type 2 diabetes, we corrected for these factors in our analysis by ANCOVA analysis. Bonferroni post hoc test was applied to correct for multiple testing. We report here the *p*-values obtained after corrections for these two confounding factors. The correlation between variables was assessed by linear regression analysis using the stepwise method. A *p* value ≤0.05 was considered statistically significant. Graphs were designed using Graphpad Prism 5.0 version for Windows (Graphpad Software, La Jolla, California, USA).

## Results

### Neutrophil serine proteases plasma concentrations in patients with liver steatosis and T2DM versus obese and lean healthy individuals

In total, PR3, NE and AAT plasma concentrations were measured and compared in four groups: 1) lean healthy control group, 2) obese (without steatosis) control group, 3) liver steatosis group and 4) type 2 diabetes group (T2DM). No statistical differences in gender distribution were observed between these groups. Seven of the obese individuals without liver steatosis and 23 of the individuals with liver steatosis had type 2 diabetes. In order to see if the differences observed between these two groups were independent of the presence of type 2 diabetes we additionally corrected for the presence of this disease when comparing them; however, p-values did not change after correction. Patients’ characteristics of all groups are shown in Table 1. Transformed data is available in Additional file 2: Table S1.

**Table 1:** Characteristics of our 4 groups of patients and controls

Variable	Lean controls (*n* = 205)	Obese controls (*n* = 105)	Liver steatosis (*n* = 166)	Type 2 diabetes (*n* = 401)
Gender(M/F)	99/106	56/49	93/73	237/164
Age (years)	33 ± 14	66 ± 5	67 ± 5	69 ± 10
BMI (kg/m^2^)	23.17 ± 2.9	30.02 ± 2.81	31.09 ± 3.6	32.62 ± 6.5
HbA1c (mmol/mol)	NA	39.41 ± 4.74	42.8 ± 8.6	63.1 ± 14.13
Liver fat content (%)	NA	2.5 ± 1.3	18 ± 13	NA

Data is expressed as mean ± SD. *BMI* body mass index, *HbA1c* glycated hemoglobin A1c, *NA* not applicable

#### NSPs and AAT concentrations in patients with liver steatosis

**Fig. 1:**
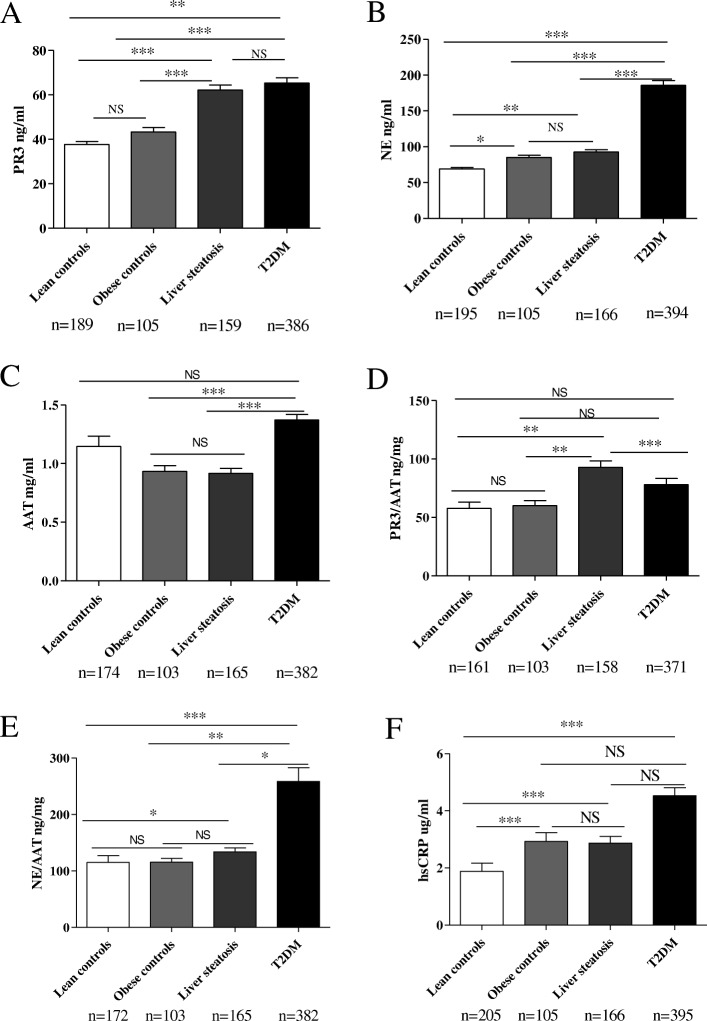
PR3, NE, AAT and hsCRP plasma concentrations in patients with liver steatosis and type 2 diabetes versus lean and obese controls. **a** PR3 plasma concentrations in patients with liver steatosis and type 2 diabetes versus lean and obese controls. **b** NE plasma concentrations in patients with liver steatosis and type 2 diabetes versus lean and obese controls. **c** AAT plasma concentrations in patients with liver steatosis and type 2 diabetes versus lean and obese controls. **d** PR3 to AAT ratio in patients with liver steatosis and type 2 diabetes versus lean and obese controls. **e** NE to AAT ratio in patients with liver steatosis and type 2 diabetes versus lean and obese controls. **f** hsCRP concentrations in patients with liver steatosis and type 2 diabetes versus lean and obese controls. Data is represented as mean ± SEM. ^*^p < 0.05, ^**^p < 0.01, ^***^p < 0.001, NS = *p* > 0.05

Plasma PR3 concentrations were significantly higher in patients with liver steatosis (*p* < 0.0001) when compared to lean and obese healthy controls (Fig. 1a) whereas PR3 concentrations were similar in lean versus obese healthy controls (Fig. 1a).

NE plasma concentrations were significantly higher in the liver steatosis group when compared to lean healthy controls group (*p* = 0.001) (Fig. 1b). NE plasma concentrations were also significantly higher (*p* = 0.019) in the obese controls group when compared to lean healthy controls group (Fig. 1b). No difference was observed between NE plasma concentrations in the liver steatosis group when compared to the obese controls group.

Regarding AAT, there was no difference in plasma concentrations between the patients with liver steatosis and obese healthy controls, while AAT concentrations tended to be higher in the lean controls group (Fig. 1c).

Furthermore, we calculated PR3 to AAT and NE to AAT ratio in order to assess the imbalance between NSPs and their natural inhibitor in patients versus controls. Interestingly, there was an increase in the PR3/AAT ratio in patients with liver steatosis versus control groups (*p* = 0.001) (Fig. 1d) while NE/AAT ratio was increased in the liver steatosis group only when compared to lean healthy controls group (0.02) (Fig. 1e). Of note, PR3 and NE correlated with each other (Additional file 3: Table S2).

Both PR3 and NE concentrations positively correlated with hsCRP concentrations (*p*<0.0001) (Additional file 3: Table S2) confirming that both PR3 and NE are released during inflammatory status and might contribute to it. As expected, hsCRP concentrations were significantly lower in the lean control group when compared to the other groups (*p* < 0.0001) (Fig. 1f).

Together, this data shows that PR3 and NE concentrations are increased in obesity while AAT concentrations are decreased. Moreover, PR3 plasma concentrations seem to be associated with the presence of liver steatosis in our cohort.

#### NSPs and AAT concentrations in patients at risk to develop ALD

In our cohort of obese individuals, some of them had increased alcohol consumption as defined by EASL (European Association for the study of the Liver) guidelines: > 30 g/day for men and > 20 g/day for women (European Association for the Study of the L, European Association for the Study of D, European Association for the Study of O 2016). Therefore, we divided this cohort in individuals at risk to develop NAFLD and individuals at risk to develop ALD (alcoholic liver disease) based on their alcohol consumption. Furthermore, based on the ^1^H-MRS fat content we subdivided these groups in individuals with liver steatosis and obese controls. PR3 concentrations were significantly higher in individuals with liver steatosis when compared to obese controls in both NAFLD risk group (*p*<0.0001) (Additional file 4: Figure S2A) and ALD risk group (*p*<0.0001) (Additional file 4: Figure S2B). No difference was observed for NE and AAT plasma concentrations between these groups (Additional file 4: Figure S2C-F). All this data suggest that PR3 is associated with the presence of liver steatosis regardless the etiology of liver injury.

#### PR3 and NE concentrations in the liver are higher in advanced histological grades of NAFLD

**Fig. 2:**
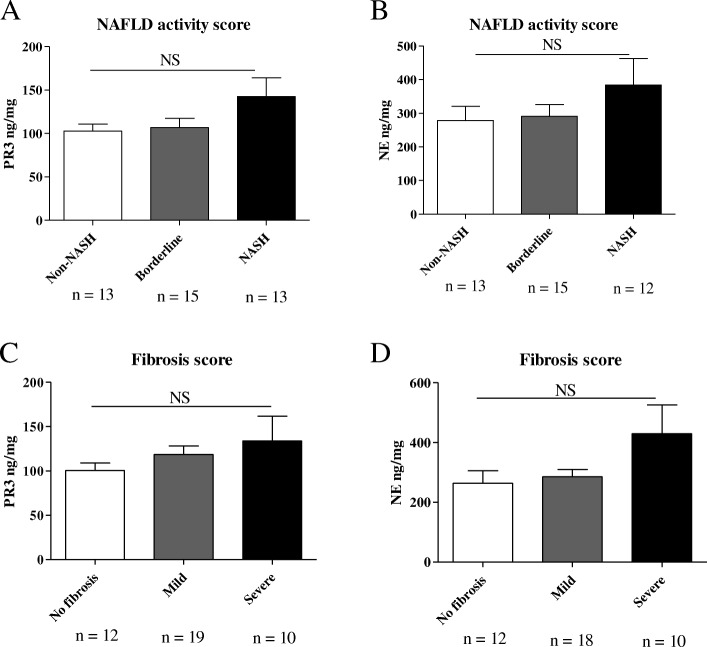
PR3 and NE liver concentrations in patients biopsy-diagnosed with NAFLD**.** Assessment of NAFLD progression: patients with a NAFLD score (NAS) (Brunt et al. 2011) between 0 and 2 were considered without NASH, patients with a NAFLD score between 3 and 4 were considered borderline and patients with a score ≥ 5 were diagnosed with NASH. Assessment of fibrosis stage (Brunt scale): patients with a fibrosis score of 1–2 were considered as having mild fibrosis and patients with a fibrosis score of 3–4 were considered as having severe fibrosis. **a** PR3 concentrations in the liver of patients with different NAS. **b** NE concentrations in the liver of patients with different NAS. **c** PR3 concentrations in the liver of patients with different fibrosis scores. **d** NE concentrations in the liver of patients with different fibrosis scores. Data is represented as mean ± SEM. NS= *p* > 0.05

To investigate whether NSPs concentrations correlated with NAFLD disease severity, we measured proteinase-3 and neutrophil elastase concentrations in liver lysates of 41 patients with different degrees of NAFLD severity. General characteristics of these patients are present in Table 2. Although not statistically significant, both PR3 and NE concentrations in the liver were higher in advanced stages of NAFLD (Fig. 2a, b). Regarding liver fibrosis, a clear trend was observed between increasing PR3 concentrations in the liver and fibrosis severity (Fig. 2c). A similar trend was observed for NE as well (Fig. 2d). Transformed data is shown in Additional file 5: Table S3.

**Table 2:** Characteristics of the 41 patients with biopsy-diagnosed NAFLD

Variable	
Gender(M/F)	23/18
Age (years)	50 ± 11
BMI (kg/m^2^)	31.23 ± 5.76

Data is expressed as mean ± SD. *BMI* body mass index

These results suggest that PR3 and NE are associated with the progression from NAFLD to NASH and liver fibrosis. Further studies, including more samples are warranted to clarify the role of NSPs in non-alcoholic liver disease development and progression.

#### NSPs and AAT concentrations in patients with type 2 diabetes

Both PR3 and NE plasma concentrations were significantly higher in patients with type 2 diabetes when compared to lean healthy controls (*p* = 0.005) and obese controls (*p* < 0.0001) (Fig. 1a, b).

Regarding AAT - plasma concentrations were significantly higher in individuals with type 2 diabetes when compared to obese individuals (*p* = 0.002) and individuals with liver steatosis (*p* < 0.0001) (Fig. 1c). Interestingly, there was no difference in PR3/AAT ratio in patients with type 2 diabetes when compared to both control groups (Fig. 1d). However, PR3/AAT ratio was significantly lower in patients with type 2 diabetes when compared to patients with liver steatosis (*p* < 0.0001) (Fig. 1d) suggesting a potential specific association of PR3 with liver steatosis. Of most importance, NE/AAT ratio was significantly increased in patients with type 2 diabetes when compared to both lean (*p* < 0.0001) and obese (*p* = 0.004) controls but also when compared to patients with liver steatosis (*p* = 0.024) (Fig. 1e).

Taken together, these results suggest that NE concentrations are up-regulated in our cohort of patients with type 2 diabetes.

**Fig. 3:**
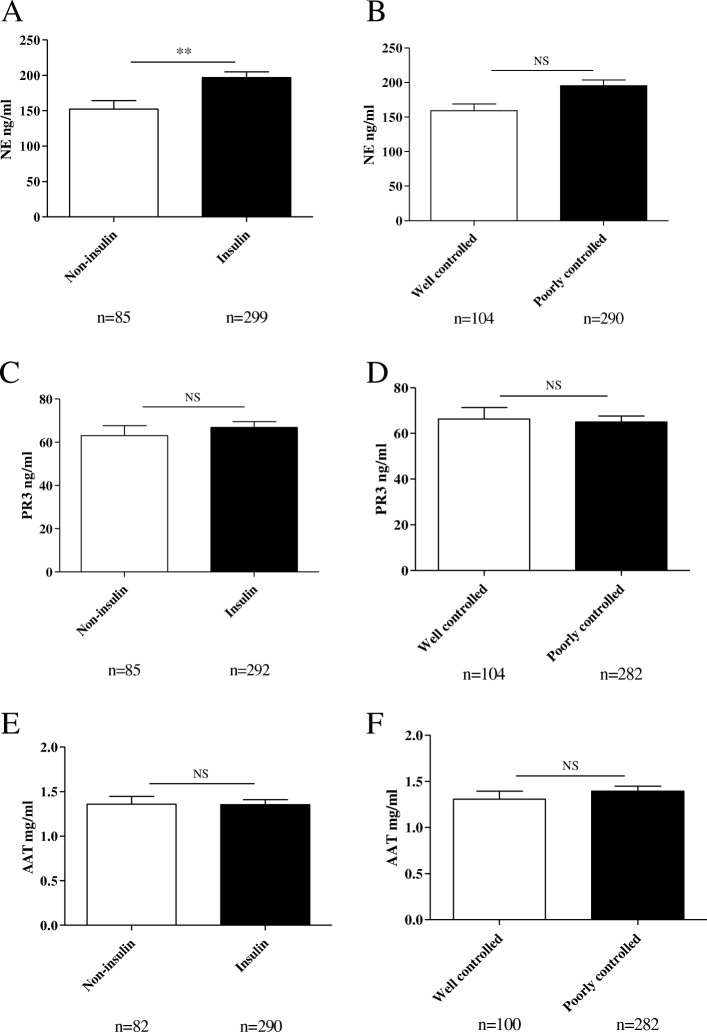
PR3, NE and AAT plasma concentrations in patients with type 2 diabetes. **a** NE plasma concentrations in patients that didn't use insulin drugs versus patients that also used insulin. **b** NE plasma concentrations between patients with well controlled glycaemia versus patients with a poor control of glycaemia. **c** PR3 plasma concentrations in patients that didn't use insulin versus patients that also used insulin. **d** PR3 plasma concentrations between patients with well controlled glycaemia versus patients with a poor control of glycaemia. **e** AAT plasma concentrations in patients that didn't use insulin versus patients that also used insulin. **f** AAT plasma concentrations between patients with well controlled glycaemia versus patients with a poor control of glycaemia. Data is represented as mean ± SEM. ^**^p < 0.01, NS= *p* > 0.05

In order to investigate this further, we tried to divide our T2DM cohort based on parameters that could indirectly reflect their degree of insulin sensitivity. First, we divided the cohort into two groups, those receiving insulin and those receiving anti-diabetes treatment other than insulin. NE plasma concentrations were significantly lower (*p* = 0.003) in patients that didn’t use insulin versus patients that were also using insulin (Fig. 3a). Since patients that use insulin probably have a higher degree of insulin resistance these findings suggest that NE concentrations in plasma could associate with the levels of insulin resistance. Second, we divided the patients based on glucose control as reflected by their HbA1c (glycated hemoglobin A1c) levels. Using a cutoff level of 53 mmol/mol HbA1c (Inzucchi et al. 2015) patients were divided in “well controlled” and “poorly controlled”. In line with our previous observation, NE plasma concentrations tended to be higher in the poorly controlled patient group when compared to the well-controlled patient group (*p* = 0.084) (Fig. 3b) while PR3 and AAT concentrations were similar in these groups (Fig. 3c-f). Transformed data is available in Additional file 6: Table S4.

These results suggest that NE plasma concentrations are able to reflect – at least partly -the level of insulin resistance in this type 2 diabetes cohort.

## Discussion

This study extends earlier findings from animal experiments and provides evidence for a role of PR3 and NE in the inflammatory states associated with type 2 diabetes in general and with NAFLD in particular in human subjects. This conclusion is based on the fact that PR3 and NE plasma concentrations are elevated both in individuals with liver steatosis and in patients with type 2 diabetes when compared to lean healthy individuals and obese individuals and are associated with hsCRP, the marker of systemic inflammation. Moreover, both PR3 and NE concentrations in the liver tended to be higher in patients with advanced stages of NAFLD when compared to patients with mild disease.

Our results, indicating that NSPs are involved in NAFLD and T2DM, are in line with previous reports. Mansuy-Aubert and co-workers elegantly showed that increased NE activity and decreased AAT serum concentrations contribute to the development of obesity, insulin resistance and liver steatosis in animal models (Mansuy-Aubert et al. 2013). Moreover, they showed an imbalance between AAT serum concentrations and NE activity in human obese subjects (Mansuy-Aubert et al. 2013). Another study, performed by Zang and co-workers, showed an increased concentration of NE and a decreased concentration of AAT in the serum of NAFLD patients when compared to healthy individuals. Additionally, they showed an increased NE/AAT ratio in the advanced stages of NAFLD with a good sensitivity and specificity to predict NASH (Zang et al. 2016). Besides the role of NE in the development of obesity-induced NAFLD and insulin resistance, our group has shown an important role for PR3 as well in these conditions in a mouse model of high-fat- diet-induced obesity (Toonen et al. 2016). In the present study we show for the first time a role for PR3 in NAFLD and type 2 diabetes in human subjects. Moreover, we show increased ratios PR3/AAT, respectively NE/AAT in the liver steatosis and type 2 diabetes groups when compared to the control groups, suggesting a deficiency in the production of AAT as a response to PR3, respectively NE plasma concentrations in these conditions. Of note, AAT concentrations were similar between individuals with type 2 diabetes and lean healthy individuals. This increase in AAT concentrations in obese individuals with type 2 diabetes could be related to the high concentrations of hsCRP that can up-regulate AAT production (Ottaviani et al. 2011). All together, our study shows that an imbalance between NSPs concentrations and their natural inhibitor, AAT, is present in obesity-associated metabolic conditions.

A limitation of our study was the lack of liver biopsies in our cohort of 271 obese individuals and a relatively small number of patients with biopsy-diagnosed NAFLD. Moreover, type 2 diabetes patients have also an increased risk to develop NAFLD, so liver biopsies in our cohort of 401 type 2 diabetes patients could have helped us differentiate the NSPs concentrations in T2DM alone versus T2DM that associate NAFLD and understand better the variation of NSPs in these two diseases. Further studies, using a larger cohort of patients with biopsy-diagnosed NAFLD, comparing not only NSPs concentrations but also enzymatic activity in plasma and liver, would help elucidate the role of these proteases in NAFLD. It would be also interesting to assess whether NSPs concentration in plasma or NSPs to AAT ratios could reflect the advanced stages of NAFLD and could be used as non-invasive markers for advanced stages of the disease.

Since PR3 and NE are able to activate cytokines and modulate the immune response (Pham 2006) they might play an important role in the development of inflammation in NAFLD and the progression to NASH. Also, due to the fact that they are able to process extracellular matrix and activate pro-fibrotic cytokines such as IL-1β and IL-33 (Interleukin-33) (Gieling et al. 2009; Marvie et al. 2010), PR3 and NE might play an important role in the mechanisms of liver fibrosis. All these characteristics make PR3 and NE potential candidates as therapeutic targets in NAFLD. One potential therapeutic agent in this respect could be alpha-1 antitrypsin.

## Conclusion

In this study we show that both PR3 and NE plasma concentrations are associated to obesity-induced metabolic disorders. Further studies are needed to explore if these proteases could be used as non-invasive markers for NAFLD and/or type 2 diabetes.

## Additional files

Additional file 1: **Figure S1.** PR3 concentrations measured in plasma samples versus serum samples. (PPTX 65 kb)

Additional file 2: **Table S1.** Natural logarithm transformed data in our four groups of patients and controls. (DOCX 13 kb)

Additional file 3: **Table S2.** Linear regression analysis for NSPs and hsCRP. (DOCX 12 kb)

Additional file 4: **Figure S2.** Levels in patients at risk to develop NAFLD or ALD. (PPTX 270 kb)

Additional file 5: **Table S3.** Natural logarithm transformed data for NAFLD stages analysis. (DOCX 12 kb)

Additional file 6: **Table S4.** Natural logarithm transformed data in our type 2 diabetes cohort analysis. (DOCX 12 kb)

## Abbreviations

^1^H-MRS: Proton magnetic resonance spectroscopy
AAT: Alpha-1 antitrypsin
ALD: Alcoholic liver disease
BCA: Bicinchoninic acid protein assay
BMI: Body mass index
EASL: European Association for the study of the Liver
ELISA: Enzyme-linked immunosorbent assay
HbA1c: Glycated hemoglobin A1c
hsCRP: High-sensitive C-Reactive Protein
IL-1β: Interleukin-1β
IL-33: Interleukin-33
NAFLD: Non-alcoholic fatty liver disease
NASH: Non-alcoholic steatohepatitis
NE: Neutrophil elastase
NSPs: Neutrophil serine proteases
PR3: Proteinase-3
T2DM: Type 2 diabetes
