# Supplementary figures and images for: Increased proteinase 3 and neutrophil elastase plasma concentrations are associated with non-alcoholic fatty liver disease (NAFLD) and type 2 diabetes
Source: Mol Med. 2019 May 2;25:16. doi: 10.1186/s10020-019-0084-3 (PMC6498541; doi:10.1186/s10020-019-0084-3)

## Slide 1
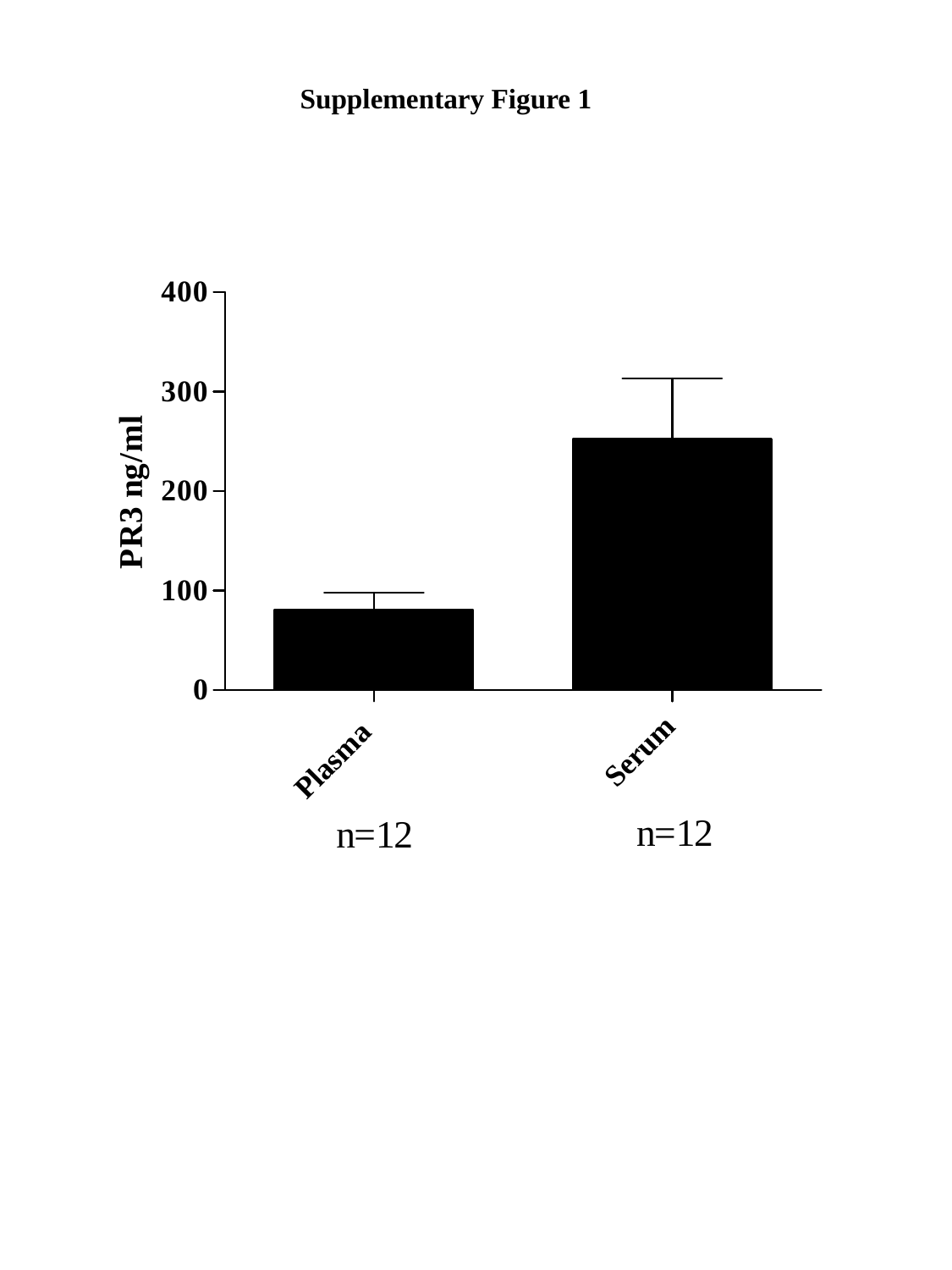

Supplementary Figure 1

Supplement: Supplementary file 1 — Figure S1. PR3 concentrations measured in plasma samples versus serum samples. (PPTX 65 kb) [file 10020_2019_84_MOESM1_ESM.pptx]
