# Supplementary material for: Increased proteinase 3 and neutrophil elastase plasma concentrations are associated with non-alcoholic fatty liver disease (NAFLD) and type 2 diabetes
Source: Mol Med. 2019 May 2;25:16. doi: 10.1186/s10020-019-0084-3 (PMC6498541; doi:10.1186/s10020-019-0084-3)
Supplement: Supplementary file 2 — Table S1. Natural logarithm transformed data in our four groups of patients and controls. (DOCX 13 kb) [file 10020_2019_84_MOESM2_ESM.docx]

**Supplementary Table 1. Natural logarithm transformed data in our four groups of patients and controls.**

| **Variable** | **Lean controls** | **Obese controls** | **Liver steatosis** | **Type 2 diabetes** |
| --- | --- | --- | --- | --- |
| **BMI (kg/m²)** | **3.13 ± 0.12** | **3.39 ± 0.08** | **3.43 ± 0.1** | **3.46 ± 0.19** |
| **PR3 ng/ml** | **3.52 ± 0.45** | **3.64 ± 0.59** | **4.04 ± 0.41** | **3.94 ± 0.69** |
| **NE ng/ml** | **4.16 ± 0.38** | **4.38 ± 0.34** | **4.44± 0.4** | **5.01± 0.63** |
| **AAT mg/ml** | **-0.19 ± 0.77** | **-0.19 ± 0.51** | **-0.24 ± 0.56** | **0.09 ± 0.72** |
| **PR3/AAT ng/mg** | **3.68 ± 0.85** | **3.85 ± 0.76** | **4.29 ± 0.69** | **3.85 ± 0.98** |
| **NE/AAT ng/mg** | **4.36 ± 0.87** | **4.59 ± 0.55** | **4.69 ± 0.64** | **4.92 ± 1** |
| **hsCRP ug/ml** | **-0.34 ± 1.34** | **0.61 ± 0.98** | **0.65 ± 0.88** | **0.82 ± 1.2** |

Data is expressed as mean ± SD. BMI, body mass index; PR3, proteinase-3; NE, neutrophil elastase; AAT, alpha-1 antitrypsin; hsCRP, high-sensitive C reactive protein.
