# Supplementary material for: Increased proteinase 3 and neutrophil elastase plasma concentrations are associated with non-alcoholic fatty liver disease (NAFLD) and type 2 diabetes
Source: Mol Med. 2019 May 2;25:16. doi: 10.1186/s10020-019-0084-3 (PMC6498541; doi:10.1186/s10020-019-0084-3)
Supplement: Supplementary file 3 — Table S2. Linear regression analysis for NSPs and hsCRP. (DOCX 12 kb) [file 10020_2019_84_MOESM3_ESM.docx]

**Supplementary Table 2. Linear regression analysis for NSPs and hsCRP.**

| \|  \|  \| **PR3** \|  \|  \|  \| **NE** \|  \|  \| \| --- \| --- \| --- \| --- \| --- \| --- \| --- \| --- \| --- \| \| **Variables** \| **R²** \| **p-value** \| **Beta** \| **p-value** \| **R²** \| **p-value** \| **Beta** \| **p-value** \| \| **NE/PR3** \| **0.169** \| **<0.0001** \| **0.14** \| **<0.0001** \| **0.169** \| **<0.0001** \| **0.128** \| **0.001** \| \| **hsCRP** \| **0.179** \| **<0.0001** \| **0.153** \| **<0.0001** \| **0.188** \| **<0.0001** \| **0.098** \| **0.013** \| |  |  |  |  |  |  |  |  |
| --- | --- | --- | --- | --- | --- | --- | --- | --- | --- | --- | --- | --- | --- | --- | --- | --- | --- | --- | --- | --- | --- | --- | --- | --- | --- | --- | --- | --- | --- | --- | --- | --- | --- | --- | --- | --- | --- | --- | --- | --- | --- | --- | --- | --- |
