# Supplementary material for: Increased proteinase 3 and neutrophil elastase plasma concentrations are associated with non-alcoholic fatty liver disease (NAFLD) and type 2 diabetes
Source: Mol Med. 2019 May 2;25:16. doi: 10.1186/s10020-019-0084-3 (PMC6498541; doi:10.1186/s10020-019-0084-3)
Supplement: Supplementary file 4 — Figure S2. Levels in patients at risk to develop NAFLD or ALD. (PPTX 270 kb) [file 10020_2019_84_MOESM4_ESM.pptx]

## Slide 1
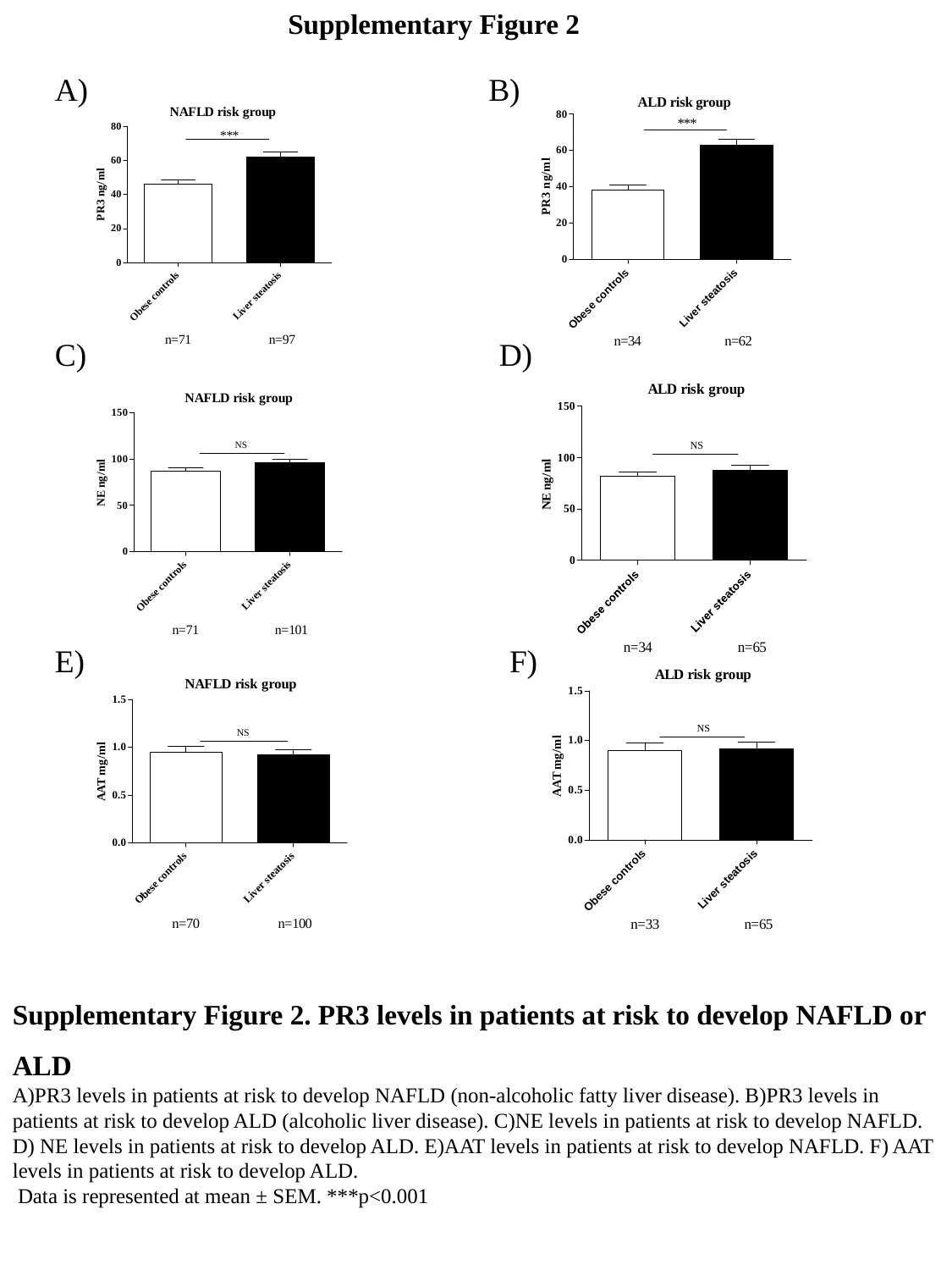

Supplementary Figure 2
A)
B)
C)
D)
E)
F)
Supplementary Figure 2. PR3 levels in patients at risk to develop NAFLD or ALD
A)PR3 levels in patients at risk to develop NAFLD (non-alcoholic fatty liver disease). B)PR3 levels in patients at risk to develop ALD (alcoholic liver disease). C)NE levels in patients at risk to develop NAFLD. D) NE levels in patients at risk to develop ALD. E)AAT levels in patients at risk to develop NAFLD. F) AAT levels in patients at risk to develop ALD.
 Data is represented at mean ± SEM. ***p<0.001
