# Supplementary material for: Increased proteinase 3 and neutrophil elastase plasma concentrations are associated with non-alcoholic fatty liver disease (NAFLD) and type 2 diabetes
Source: Mol Med. 2019 May 2;25:16. doi: 10.1186/s10020-019-0084-3 (PMC6498541; doi:10.1186/s10020-019-0084-3)
Supplement: Supplementary file 5 — Table S3. Natural logarithm transformed data for NAFLD stages analysis. (DOCX 12 kb) [file 10020_2019_84_MOESM5_ESM.docx]

|  | Parameter | PR3 | NE |
| --- | --- | --- | --- |
|  | **Non-NASH** | **4.6 ± 0.28** | **5.5 ± 0.48** |
| NAFLD score | **Borderline** | **4.61 ± 0.34** | **5.5 ± 0.45** |
|  | **NASH** | **4.8 ± 0.46** | **5.8 ± 0.52** |
|  | **No fibrosis** | **4.57 ± 0.26** | **5.45 ± 0.5** |
| Fibrosis score | **Mild** | **4.71 ± 0.36** | **5.58 ± 0.39** |
|  | **Severe** | **4.77 ± 0.47** | **5.89 ± 0.58** |

**Supplementary Table 3. Natural logarithm transformed data for NAFLD stages analysis.**

Data is expressed as mean ± SD. PR3, proteinase-3; NE, neutrophil elastase; NAFLD, non- alcoholic fatty liver disease.
