# Supplementary material for: Increased proteinase 3 and neutrophil elastase plasma concentrations are associated with non-alcoholic fatty liver disease (NAFLD) and type 2 diabetes
Source: Mol Med. 2019 May 2;25:16. doi: 10.1186/s10020-019-0084-3 (PMC6498541; doi:10.1186/s10020-019-0084-3)
Supplement: Supplementary file 6 — Table S4. Natural logarithm transformed data in our type 2 diabetes cohort analysis. (DOCX 12 kb) [file 10020_2019_84_MOESM6_ESM.docx]

**Supplementary Table 4. Natural logarithm transformed data in our type 2 diabetes cohort analysis.**

| **Variable** | **Oral medication** | **Insulin** | **Well controlled** | **Poorly controlled** |
| --- | --- | --- | --- | --- |
| **NE** | **4.83 ± 0.58** | **5.07 ± 0.64** | **4.92 ± 0.52** | **5.05 ± 0.66** |
| **PR3** | **3.94 ± 0.64** | **3.96 ± 0.71** | **3.92 ± 0.75** | **3.95 ± 0.67** |
| **AAT** | **0.1 ± 0.71** | **0.05± 0.82** | **0.008 ± 0.8** | **0.09 ± 0.8** |

Data is expressed as mean ± SD. NE, neutrophil elastase; PR3, proteinase-3; AAT, alpha-1 antitrypsin.
